# Computational Analysis of Pathological Image Enables Interpretable Prediction for Microsatellite Instability

**DOI:** 10.3389/fonc.2022.825353

**Published:** 2022-07-22

**Authors:** Jin Zhu, Wangwei Wu, Yuting Zhang, Shiyun Lin, Yukang Jiang, Ruixian Liu, Heping Zhang, Xueqin Wang

**Affiliations:** ^1^ Southern China Center for Statistical Science, School of Mathematics, Sun Yat-Sen University, Guangzhou, China; ^2^ Center for Statistical Science, School of Mathematical Sciences, Peking University, Beijing, China; ^3^ Department of Clinical Laboratory, The Sixth Affiliated Hospital of Sun Yat-Sen University, Guangzhou, China; ^4^ School of Public Health, Yale University, New Haven, CT, United States; ^5^ Department of Statistics and Finance/International Institute of Finance, School of Management, University of Science and Technology of China, Hefei, China

**Keywords:** cancer, microsatellite instability, interpretability, deep learning, random forest

## Abstract

**Background:**

Microsatellite instability (MSI) is associated with several tumor types and has become increasingly vital in guiding patient treatment decisions; however, reasonably distinguishing MSI from its counterpart is challenging in clinical practice.

**Methods:**

In this study, interpretable pathological image analysis strategies are established to help medical experts to identify MSI. The strategies only require ubiquitous hematoxylin and eosin–stained whole-slide images and perform well in the three cohorts collected from The Cancer Genome Atlas. Equipped with machine learning and image processing technique, intelligent models are established to diagnose MSI based on pathological images, providing the rationale of the decision in both image level and pathological feature level.

**Findings:**

The strategies achieve two levels of interpretability. First, the image-level interpretability is achieved by generating localization heat maps of important regions based on deep learning. Second, the feature-level interpretability is attained through feature importance and pathological feature interaction analysis. Interestingly, from both the image-level and feature-level interpretability, color and texture characteristics, as well as their interaction, are shown to be mostly contributed to the MSI prediction.

**Interpretation:**

The developed transparent machine learning pipeline is able to detect MSI efficiently and provide comprehensive clinical insights to pathologists. The comprehensible heat maps and features in the intelligent pipeline reflect extra- and intra-cellular acid–base balance shift in MSI tumor.

## Introduction

Microsatellite instability (MSI) is the condition of genetic hypermutability that results from impaired DNA mismatch repair. Cells with abnormally functioning mismatch repair are unable to correct errors that occur during DNA replication and consequently accumulate errors. MSI has been frequently observed within several types of cancer, most commonly in colorectal, endometrial, and gastric adenocarcinomas (1). The clinical significance of MSI has been well described in colorectal cancer (CC), as patients with MSI-high colorectal tumors have been shown to have improved prognosis compared with those with MSS (microsatellite stable) tumors (2). In 2017, the U.S. Food and Drug Administration approved anti–programmed cell death-1 immunotherapy for mismatch repair deficiency/MSI-high refractory or metastatic solid tumors, making the evaluation of DNA mismatch repair deficiency an important clinical task. However, in clinical practice, not every patient is tested for MSI, because this requires additional next-generation sequencing (3, 4), polymerase chain reaction (5), or immunohistochemical tests (1, 6, 7). Thus, it is in high demand for a cheap, effective, and convenient classifier to assist experts in distinguishing MSI vs. MSS.

Numerous publications have identified histologic features that are more commonly seen in MSI. By far, it is a well-known fact that tumors that have undifferentiated morphology, poor differentiation, and the high infiltration of TIL cells are more likely to be MSI (8–11). Unfortunately, it is still challenging to distinguish MSS from MSI based on pathologist’s visual inspections from pathological images because the morphology of MSS is similar to that of MSI (12). The recent technical development of high-throughput whole-slide scanners has enabled effective and fast digitalization of histological slides to generate WSIs. More importantly, the thriving of various machine learning (ML) methods in image processing makes this task accessible. In recent years, ML has been broadly deployed as a diagnostic tool in pathology (13, 14). For example, Iizuka et al. built up convolutional neural networks (CNNs) and recurrent neural networks to classify WSI into adenocarcinoma, adenoma, and non-neoplastic (15). The study by Bar et al. demonstrated the efficacy of the computational pathology framework in the non-medical image databases by training a model in chest pathology identification (16). Notably, deep learning (DL) model has been used to predict MSI directly from H&E histology and reported the network achieved desirable performance in both gastric stomach adenocarcinoma (STAD) and CC (17). These studies attest to the great potential of ML methods in medical research and clinical practice.

There is no doubt that the ML revolution has begun, but the lack of the “interpretability” of ML is of particular concern in healthcare (18, 19). Here, the “interpretability” means that clinical experts and researchers can understand the logic of decision or prediction produced by ML methods (20). In essence, it urges ML systems to follow a fundamental tenet of medical ethics, that is, the disclosure of necessary yet meaningful details about medical treatment to patients (21). Unfortunately, to the best of our knowledge, most of the existing MSI diagnosis systems, especially DL-based systems, are non-interpretable. Therefore, there is an urgent need to establish a new research paradigm in applying an interpretable ML system in medical pathology field (22–26).

In this study, we used H&E-stained WSI from TCGA: 360 formalin-fixed paraffin-embedded (FFPE) samples of CC (TCGA-CC-DX) (27), 285 FFPE samples of STAD (TCGA-STAD) (28), and 385 snap-frozen samples of CC (TCGA-CC-KR). H&E-stained images in these databases have already been tessellated into 108,020 (TCGA-STAD), 139,147 (TCGA-CC-KR), and 182,403 (TCGA-CC-DX) color-normalized tiles (17), and all of them only target region with tumor tissue. The aims of the study are as follows: (i) to build an image-based ML method on MSI classification and post-process the fed image to a heat map to interpret the diagnosis of MSI at an image level; and (ii) to design a fully transparent feature extraction pipeline and understand the pathological features’ importance and interactions for predicting MSI by training a feature-based ML model.

Our contributions are two folds. First, we developed ML models with decent power in the prediction of MSI. This model can exhibit a visual heatmap demonstrating high-contribution regions for MSI prediction in the H&E image. Second, we certified certain pathological features with non-trivial importance in MSI classification, which is not explicitly studied in the previous research. Therefore, our study facilitates MSI diagnosis based on H&E image and sheds light on the understanding of MSI at both image-level and features level.

## Materials and Methods

### Histopathology Image Sources

The whole-slide H&E-stained histopathology images were obtained from TCGA, including three cancer subtype datasets. Dataset DX consisted of 295 MSS patients and 65 MSI patients from FFPE samples of CC. Dataset KR contained 316 MSS patients and 72 MSI patients from snap-frozen samples of CC. Dataset STAD collected 225 MSS patients and 60 MSI patients of FFPE STAD. Two criteria in the published study (17) classify patients as MSI: (i) all the patients who were previously defined as MSI were included in the MSI group (29); and (ii) some patients with unknown MSI status but with a mutation count of >1,000 were also defined as MSI (30).

All the images used in our models have already gone through tumor tissue detection and have been tessellated into small tiles in J.N. Kather’s work (https://zenodo.org/record/2530835 and https://doi.org/10.5281/zenodo.2532612). The proceeding for getting the tiles is of two steps. First, the tumor region is identified from WSI image, and second, the tumor is divided into small square subregions, called tiles, where the edge of each tile is 256 µm. There are 108,020 tiles in TCGA-STAD cohort, 139,147 in TCGA-CC-KR, and 182,403 in TCGA-CC-DX. Color normalization has already been performed on every tile using the Macenko method (31), which converts all images to a reference color space. In all cases, training and test sets were split on a patient level, and no image tiles from test patients were present in any training sets.

### Details of Deep Learning and Grad-CAM

The DL model that we considered is ResNet-18, which is one of the state-of-the-art CNNs (17, 32). We adopted all of the default settings in ResNet-18 and did not fine-tune any hyperparameters on it. ResNet-18 is built in Python 3.7 with TensorFlow-GPU 1.14.0 and Keras 2.3.0. Because the ResNet-18 is insensitive to the adversarial samples, we did not pre-process any image tiles in the three TCGA datasets. The patient-level areas under the curve (AUCs), receiver operating characteristic (ROC) curves, and 95% stratified bootstrap confidence intervals (CIs) for ROC curves were computed and visualized by two R packages: pROC (33) and ggplot2 (34). Gradient-weighted Class Activation Mapping (Grad-CAM) utilizes the gradient information abundant in the last convolutional layer of a CNN and generates a rough localization map of the important regions in the image. We apply the rectified linear unit to the linear combination of maps to generate localization maps of the desired class. Grad-CAM visualization was implemented in Python 3.7 with TensorFlow-GPU 1.14.0 and Keras 2.3.0.

### Image Pretreatment

Before feature extraction, we apply pretreatments to the tiles before feature extraction and we summarized the pretreatments and associated implementation details in **Table 1**. First, white balance is performed on our cohorts because the natural appearance tone of the object may alter in the formation of images when exposed in a lightning condition of different color temperature (37). Because every tile has an area without cell organization, i.e., without H&E stain, we could view that part as the neutral reference in adjustment. In addition to the color cast, overexposure and underexposure also may result in the distortion of our features (38). Still, taking the unstained area as the reference, we regulated all tiles into the same level of brightness. In addition, to get the location of immune cells’ nuclei, we similarly perform color deconvolution (39, 40) to separate color space from immunohistochemical staining on each tile. Finally, to extract the Haralick texture features (41, 42) of tumor cells, we used a positive cell detection algorithm to locate every tumor cell in each tile and use its batch process to get needed features.

**Table 1:** Pre-treatment, software, and parameters used in each pre-treatment.

Pretreatment	Software	Parameters
White balance	OpenCV-Python	Default
Brightness Adjustment	OpenCV-Python	Target average brightness in RR: 240
Color Deconvolution	ImageJ (35)	Default
Tumor Cell Identification	scikit-image (36)	Objects with size: 5–17

Reference region (RR): an area without cell organization, whose values in RGB channels within (180, 255). The parameters are manually selected according to the experience of image analysis for H&E images.

### Feature Extraction

In global color feature extraction, the region of interest (ROI) is a stained area. We recorded mean value, quantiles (25%, 50%, and 70%), and higher-order moments (variance, kurtosis, and skewness) in ROI of each channel in RGB and HSV as our global features. Moreover, with Gaussian mixture model (GMM) model (43), we perform image segmentation to each tile to divide the ROI into three clusters and record the corresponding features in every cluster as our local features. We located immune cells’ nuclei after color deconvolution according to their size and grayscale and calculated the amount as the feature. As for the differentiation degree of tissue in tiles, we performed dilation, erosion, and circle Hough transforms (44) to identify outlines similar to circle in images and to decide their differentiation degree. Because the more regular shapes exist, the more highly the tissue differentiates. Because we have recorded the tumor cell’s location, we extract Haralick features of each tumor cell in one tile and adopt the mean value of all cells’ as this tile feature *via* QuPath software (45). In addition, we also recorded the count of a tumor cell as our feature.

### Details of Random Forest and Benchmark Machine Learning Methods

Our RF method was built and tested using Python version 3.7.1 with RandomForestClassifier in sklearn.ensemble library (46). During training, 70% of patients in every dataset were randomly selected, and all of their tiles were used in training, whereas the rest of the tiles were held out and used as test sets. There are some anomalous tiles in each dataset, i.e., blurred or color disorder, resulting in the loss of the information contained in them. Therefore, we disposed of all of them in every dataset. In addition, we also delete the tiles owning an extreme immune cell number (a value that significant in 1% level) because an extremely small number may represent the non-tumor area, whereas a too large number represents lymphatic concentration area. In each forest, we set 500 trees in total and take Gini impurity as the criterion. For each forest, we tune the minimum node size of random forest (RF), which is an important parameter to prevent overfitting, and we keep other parameters with the default settings. We used a simple tuning criterion as follows: Consider the candidate minimum node size: 15, 16, …, 25, and then the size associated with the least out-of-bag error of RF is chosen. The selected minimum node size is 23 for the STAD cohort, 17 for both the KR and the DX cohorts. Again, we used pROC packages to compute AUC and assess 95% stratified bootstrapped CIs and ggplot2 package to visualize the model performance.

Out of comparison, we also consider two benchmarking ML methods suggested by a reviewer including support vector machine (SVM) (47) and generalized linear model (GLM) (48). The ridge regularization in GLM is selected *via* 10-fold cross validation. Because hundreds of thousands of tiles brought huge computational burden, SVM ran very slow even in the state-of-the-art implementation (49), and thus, we did not tune the parameters in SVM and set them as default.

### Permutation Feature Importance and Conditional Minimal Depth

Permutation-based feature importance (50) is a widely used model inspection technique for RF. It is defined to be the decline in a model accuracy when one feature’s values are randomly shuffled. The shuffle procedure cancels the relationship between the label and the feature, and thus, the drop in the model accuracy can serve as a measurement for the importance of the feature in RF. An alternative feature importance, minimal depth (51), is defined as the depth when a feature splits for the first time in a tree. For example, if a feature splits the root node in a tree, then its minimal depth is 0. The mean of minimal depths over all trees in a forest can measure the feature importance. The importance ordering of features under it keeps highly consistent with the result from the permutation-based method (**Figure S4**).

To investigate the interaction between two different features, we used a generalization of minimal depth, conditional minimal depth, that measures the depth of the second feature in a subtree with the root node where the first feature splits (52). Specifically, we recorded all of such splits with the first feature and calculated the mean of conditional minimal depths of the second features given the first feature. A large gap between the mean of conditional minimal depth and the mean of minimal depth implies possibilities for the second feature being used for splitting after the first feature. The occurrence of the large gap implies that the two features have a strong interaction. We used R version 3.5.1 with randomForest package (53) to rebuild that RF and analyze and visualize the relations between different features with randomForestExplainer package (52).

### Ablation Experiment for Deep Learning

Ablation experiment (54–56) is conducted to investigate the contribution of pathological features in DL. Specifically, we eliminated the RGB mean differences between MSI and MSS groups in the test set by adjusting the mean value in each tile in the test set to the mean value of all the tiles as a whole. Then, we feed the adjusted tiles in the test set into the trained neural network. The drops of AUCs after reevaluation can verify the contribution of the RGB feature in the classification of the DL network.

### Role of the Funding Source

The funder of this study had no role in study design, data collection, data analysis, data interpretation, and writing of the report. The corresponding author had full access to study data and final responsibility for the decision to submit for publication.

## Results

### A Deep Learning Classifier and Image-Level Visual Interpretability

**Figure 1:**
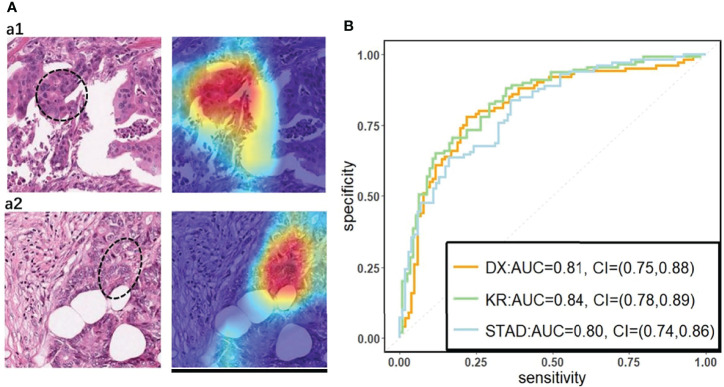
**(A)** The original tile and the corresponding heatmap output by the GCAM. The image in the left of **(A1)** and **(A2)** display tiles from the TCGA-CC-DX dataset labeled with MSI and MSS, respectively. The ellipse upon the images corresponds the most contributed region revealed by GCAM. In the heatmaps, the brighter region contributes more to the classification. For instance, the red one is the most highlighted area, while the blue regions contribute limitedly. Scale bar, 256 µm **(B)** Patient-level receiver operating characteristic (ROC) curve for classifying MSI versus MSS in the three datasets with deep learning. The 95% confidence intervals (CI) were computed by the bootstrap method.

We used a commonly used end-to-end CNN, ResNet-18 (32) in the study. To fit this DL model for different cancer subtypes, we trained three ResNet-18 networks based on 70% of the tiles randomly sampled from three datasets, the remaining 30% of the tiles in each dataset were used for testing. In the testing cohort, a patient’s slide was predicted to be MSI if at least half of the tiles were predicted to be MSI. The patient-level accuracy and AUC were 0.84 in the KR cohort, 0.81 in the DX cohort, and 0.80 in the STAD cohort (**Figure 1B**).

On the basis of the trained DL model, the Grad-CAM was used to make the convolutional-based model more transparent by generating localization maps of the important regions (57). To unveil the hidden logic behind the DL and provide visual interpretability, we deployed Grad-CAM to find out which part of the H&E image supports DL’s classification. Two typical images for interpreting DL prediction logic are shown (**Figure 1A**). The region highlighted by Grad-CAM points out the important region for DL decision but not statistical correlation. Our pathologist noted that the highlighted region in **Figure 1A** tended to be where immune cells are mainly concentrated in the tumor organism; meanwhile, we also found that the highlighted region presented distinct color and texture characteristics. We were intrigued by this phenomenon and further examined this important region in great detail.

### Transparent Pathological Image Analysis Workflow and Feature-Based Classification Model

**Figure 2:**
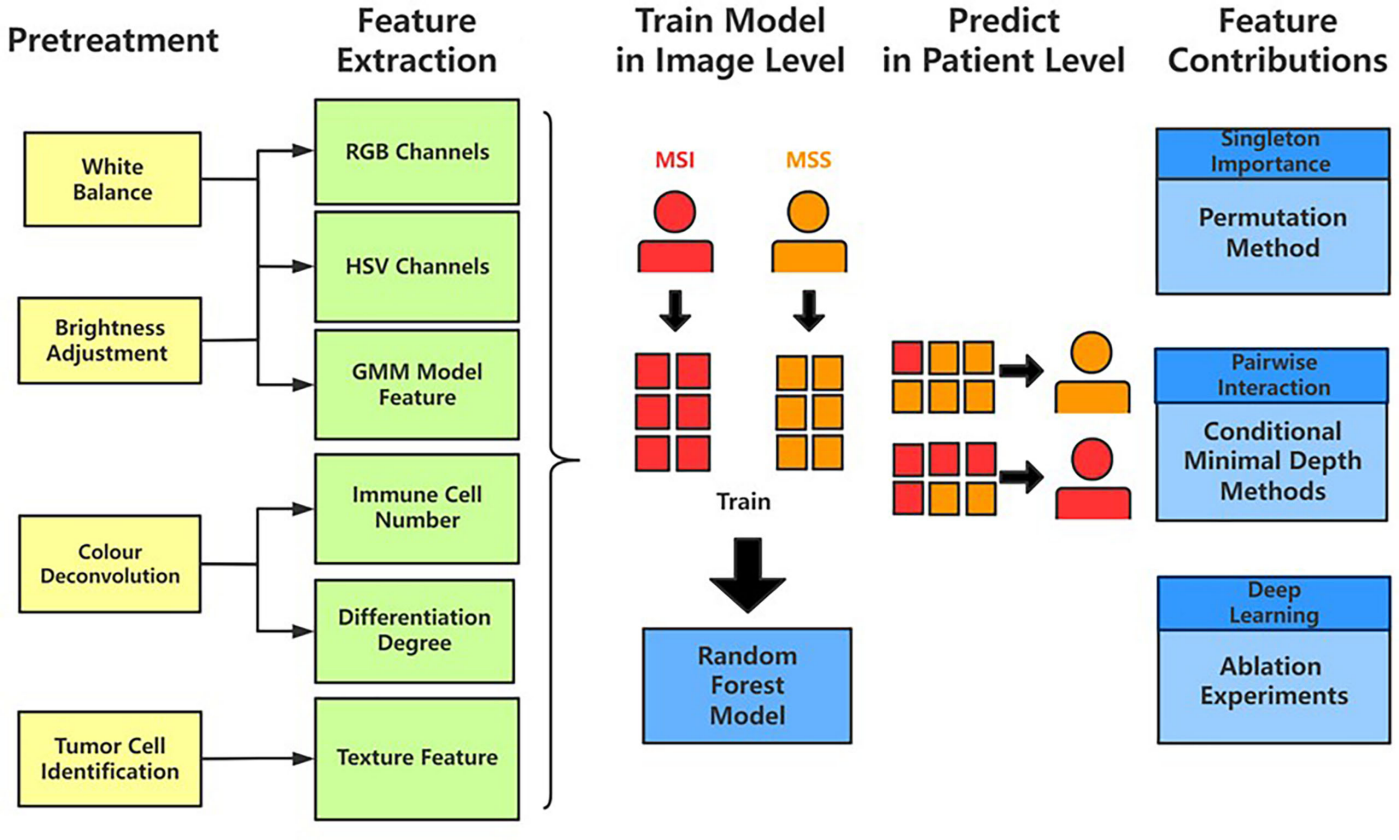
The workflow of studying pathological features in discriminating against MSI from MSS. Five main steps—pretreatments, feature extraction, model training, patient-level predictions, and feature contributions analysis—were sequentially executed to improve image quality, generate pathological features, build statistical model, evaluate model performance, and measure features’ contributions, respectively.

**Figure 3:**
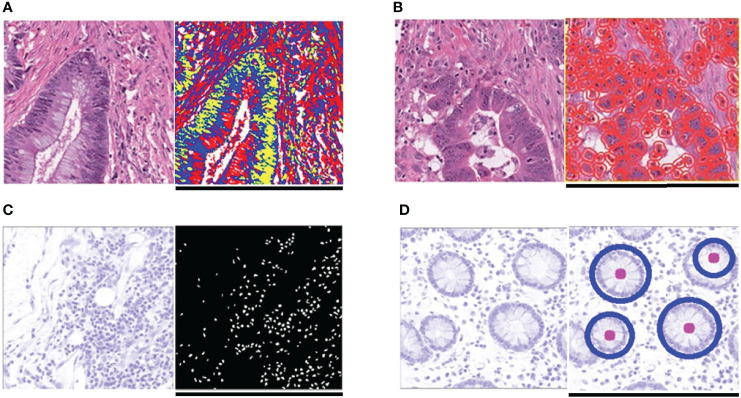
Typical feature extraction result. **(A)** GMM model for image segmentation. The figure on the left is a tile from the TCGA-CC-DX dataset, and its image segmentation tiles processed by the GMM method are shown in the figure on the right. The green part whose grayscale is the lowest among the three parts tends to be tumor tissue, whereas the blue and red ones represent non-tumor tissue. **(B)** Tumor cell detection before Haralick texture identification. The figure on the left is an original tile, whereas the one on the right is processed with tumor identification. Each red circle in the tile on the right indicates the boundary of one tumor cell. **(C)** Infiltrating immune cells detection. The detection of immune cells allows us to calculate the connectivity domain. **(D)** The grading of differentiation. Detect the circularly similar arrangement in one slice and grade the degree of differentiation based on its amount. Scale bar, 256 µm.

The results from Grad-CAM suggested that certain features of the H&E-stained images might encode essential regions of the tumor organism. To further investigate this, we developed a multi-step, automatic and transparent workflow (**Figure 2**). In the first step, we standardized the three image datasets by standard image processing techniques (e.g., white balance and brightness adjustments). After the image pre-processing, we extracted visible pathological features. Motivated by the feedback from Grad-CAM and existing studies (9, 58, 59), we focused on these H&E feature characteristics: global and local color features in RGB and HSV channels, the numbers of infiltrating immune cells and tumor cells, the grading of differentiation, and the texture features from tumor cells. A total of 182 features were extracted from each image tile, and some representative ones are displayed in **Figure 3**.

**Figure 4:**
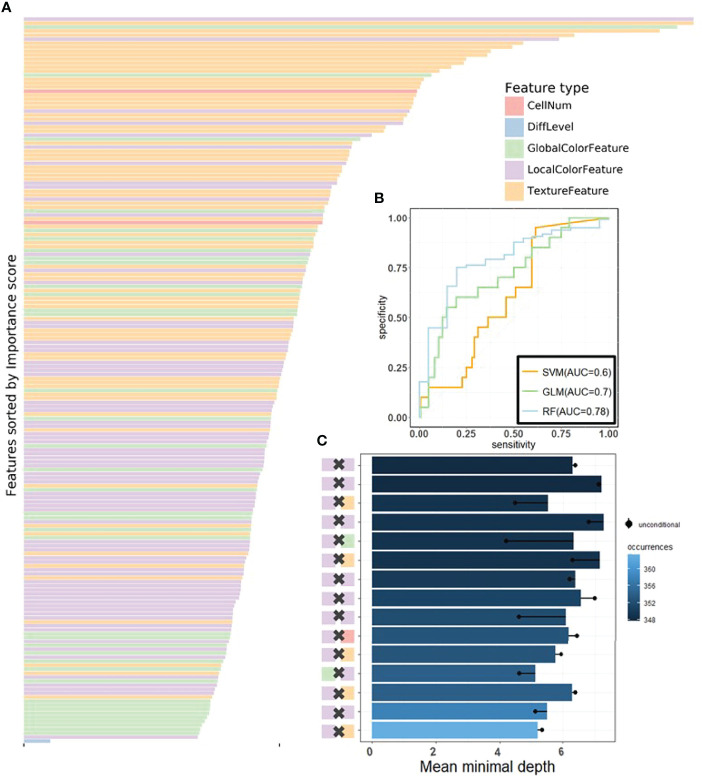
The visualization of performance and interpretability of the RF in KR dataset. **(A)** The bar plot of permutation-based variable importance. Features are arranged from top to bottom in order of importance (the names of the features are provided in the order in **Table S2**). **(B)** The patient-level ROC curve for classifying MSI versus MSS with random forest. Three colors distinguish GLM, SVM, and RF. The 95% confidence intervals (CIs) computed by the bootstrap method are as follows: (0.53, 0.83) for GLM, (0.49, 0.72) for SVM, and (0.70, 0.82) for RF. **(C)** The bar plot of the mean of conditional minimal depth (the top 15 feature pairs of interaction are shown). A feature pair of interaction is listed as A × B, where A and B are one of feature type and their concrete names are listed in **Table S3**. Feature pairs are arranged from the bottom to top in the order of the occurrences, which are represented by the color intensity of the bars. The bar’s length indicates the mean of conditional minimal depth and the distance from the dot to the y-axis measures the mean of minimal depth of **(B)** The length of the dot line implies the gap between them, measuring the effect of pairwise feature interaction. A large gap implies a strong interaction (see also **Figures S1–S3**).

We then applied RF (50), one of the most popular ML algorithms, to all three databases to classify MSI versus MSS on H&E-stained histology slides. We randomly selected 70% of patients in every dataset during training, and all their tiles were used in training, whereas the rest of the tiles were held out and used as test sets. In the test sets of each dataset, true MSS image tiles cohort had a median MSS score (the proportion of the prediction result judged to be MSS in each decision tree of the forest) that was significantly different from those of MSI tiles (the P-values of the two-tailed *t* test were 0.02, 0.0024, and 0.002 in the three datasets), indicating that our models can distinguish MSI from MSS. Because one patient may have many different tiles, we obtained the patient-level MSI scores by averaging the RF’s prediction on all its tiles. AUCs for MSI detection were 0.78 (95% CI: 0.7–0.82) in KR cohort, 0.7 (95% CI: 0.65–0.74) in DX cohort, and 0.74 (95% CI: 0.65–0.79) in STAD cohort (see **Figures 4B**, **Figures S1B, S2B**). These results show that visible pathological features can be useful in MSI prediction. Comparing the AUCs of DL and RF, we can see that DL is superior to RF in prediction, yet we would show that RF can reveal informative messages about the impact of pathological features on MSI prediction. From the comparison among RF, SVM, and GLM, we see that, from predictive power, RF surpasses the other benchmarking ML methods.

### Feature-Level Visual Interpretability: Feature Importance and Interactions

One of the attractive advantages of RF is that it can evaluate the importance of the features. Therefore, we verify and quantify these features’ power in distinguishing MSI from MSS by extracting information from a trained model. A representative pattern can be discovered from the visualization of permutation-based feature importance (50, 60) in the KR dataset (**Figure 4A**) . From the figure, we can deduce that the texture features play a dominant role. Because the texture features reflect the surface’s average smoothness of the tumor cells in one tile, we deduce that the characteristics of the tumor surface are an important clinical indicator in automatic MSI diagnosis. Color features also have important contributions. In the global color feature, the higher-order statistics (skew and kurtosis) contribute more than the first-order statistics (mean and quantile), indicating that some useful information contributing to classification are hidden in high-order features. Local color features also deserve our attention. Compared with global color features, the local ones were useful in image segmentation by dividing slices into different clusters, and we obtained the information in each cluster. **Figure 3** demonstrates the clinical utility of the clusters as they closely reflected tumor tissue versus non-tumor tissue. The number of infiltrating immune cells was also important as expected, whereas the differentiation grade contributed the least in every dataset.

**Figure 5:**
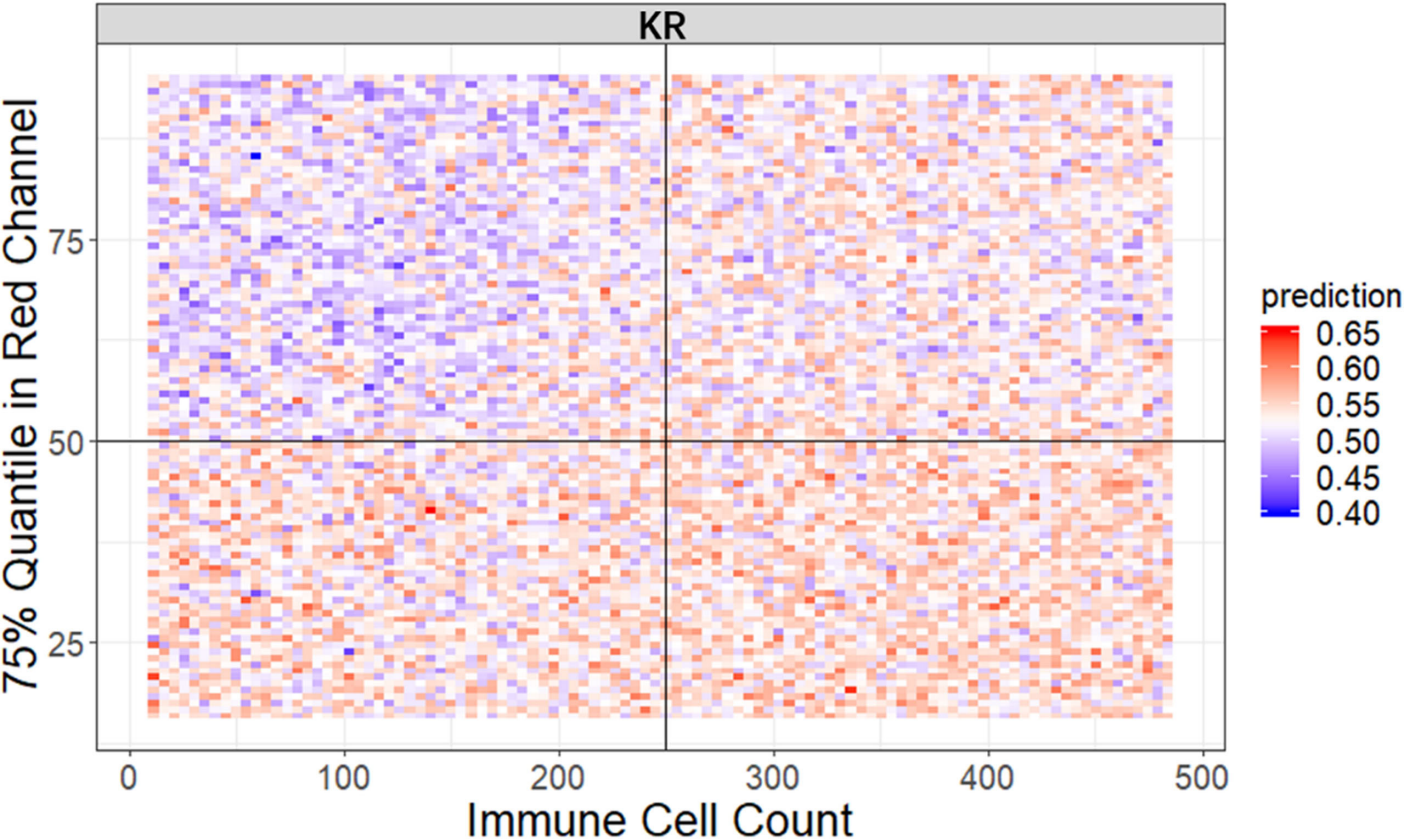
The visualization of typical pairwise features’ interaction in KR dataset. The prediction value ranges from 0 to 1 with color from blue to red. The bluer means a larger probability of MSI, whereas the redder tends to be MSS (see also **Figure S3**).

It is widely accepted that feature interactions (i.e., the joint effect of features) can be important for the complex disease (61–64). Our feature-based RF models also allow us to exploit the pairwise feature interactions in MSI classification, and thus, we can attain a more clear understanding of the characteristics of MSI tiles and the mechanism of RF. Here, we use conditional minimal depth (51) to quantitatively assess feature interaction and then demonstrate the foremost 15 pairwise interactions (**Figures 4C**, **Figures S2C, S3C**). The feature types with the most effective interaction effect with other features in each dataset are the local color feature in KR, the global color feature in DX, and texture features in STAD. The three features enhanced the importance of the features interacting with them, even the features themselves may have a weak effect before. It is also worthy to note that interactions incline to occur more often between color features and texture features or between local color and global color features. To understand how the paired features jointly help the MSI diagnosis, we plot the prediction values of typical feature interaction on a grid diagram (**Figure 5** and **Figure S3**). In the KR dataset, a greater immune cell number and a lower value of the 75th percentile of red channel lead to a higher probability of MSS. In DX, a higher value of the max caliper in tumor cells and a fewer tumor cell number lead to a higher probability of MSS. In STAD, a lower value of the optical density range of tumor cells’ nucleus in Hematoxylin stains and a higher value of texture feature correlation in eosin stains lead to a higher probability of MSS.

## Discussion

To our knowledge, this is the first study to not only build up a classification model in distinguishing MSI from MSS but also provide an interpretability analysis. Previous studies in investigating the pathologic predictors of MSI through feature extraction and logistics regression model suffered from the limited learning capability as well as the small sample size and thus could not achieve satisfactory performance (9). Other works on MSI classification paid attention to the enhancement of the prediction accuracy by establishing a DL network but did not provide a detailed description of the mechanism behind the model (17). In this study, we tackled these problems through using three different cancer types datasets from TCGA and following the framework of interpretability with two steps: first, built up a high-performance DL network with a visual explanation capacity as model-based interpretability; second, we further analyzed and confirmed features’ power using a feature-based interpretable model.

To build an interpretable DL network, we trained residual learning CNNs and deployed Grad-CAM to the final convolutional layer of the network to produce the heatmap that reflects the highly contributed region. Notably, through its coarse localization map of the image’s essential regions, it provided preliminary insight into highly contributed pathological features. It is worthy to note that the prediction performance of our method is also desirable, and it is comparable to the predictors proposed in other published research (17). Although Grad-CAM is also used in the recent literature, they just use it to quantify possible differences between real and synthetic images.

To understand the contribution of the pathological features on MSI classification, we manually extracted the clinically meaningful features *via* image processing methods, trained an RF classifier based on those features, assessed the importance of those features, and exploited their interaction. This procedure achieves feature level interpretability at the expense of prediction performance; however, we interestingly found that the texture and color of the H&E image and the interactions among them were crucial for diagnosing MSI. To the best of our knowledge, this has not been noted before. From the widely studied underlying biology of immune infiltration in MSI, numerous pieces of evidence indicate that a high tumor mutational burden increases the likelihood that immunogenic neoantigens expressed by tumor cells induce increased immune infiltration (65–67). In addition, color feature is regard as an important feature for the diagnosis of TFE3 Xp11.2 translocation renal cell carcinoma *via* WSI (58). Finally, the pivotal roles of color and texture features found in our study reflect extra- and intracellular acid–base balance shift in MSI tumor (68). Another interesting fact is that the feature type that tends to interact with the other features has a clear difference in the three datasets due to the image heterogeneity raised from the diversity of cancer type (CC or STAD) and tissue preservation methods (snap-frozen or FFPE) (69), indicating that the feature interaction mode was influenced by preservation methods and tumor types. However, this insight would not be attained from “black-box” ML method. Moreover, we hypothesized that the dominant-role features such as color in RF models were also important in the DL model. To test our hypothesis, we eliminated the mean color differences between MSI and MSS groups and reevaluated our DL models’ AUCs. Specifically, we calculated the RGB mean value of all tiles in both groups and centralized the RGB mean value of every tile into that population mean value. We found that the AUCs were reduced by 0.11, 0.12, and 0.14 in DX, KR, and STAD datasets, respectively, supporting our hypothesis that color features also contributed to the DL model.

We note that our findings warrant replications through further biological experiments. The H&E stain is capable of highlighting the fine structures of cells and tissues. Most cellular organelles and extracellular matrix are eosinophilic, whereas the nucleus, rough endoplasmic reticulum, and ribosomes are basophilic. Our study shows that the spectrum, intensity, and texture of colors matter in distinguishing MSI from MSS, which needs further validation. We hypothesize that MSI tumor usually has distinct color/texture characteristics due to diverse gene mutation pattern (1, 70). Furthermore, the methodology of this study could be applied to the pathological analysis of other diseases, like infectious, in which color/texture characteristics of the H&E images are also crucial for disease diagnosis. One limitation of this study is that the cases in TCGA datasets may not be an unbiased collection from the real situation because pathologists may only upload the representative ones. Although our model performed well in these histopathology images, we should admit that their performance in the actual clinical settings requires further research. Therefore, one of our future direction is integrating more available datasets considered in (71), and we point out that it can naturally improve the specificity and control sensitivity simultaneously. Another limitation is that our study only focused on H&E-stained images, and we could not confirm whether the pattern in this study, especially the color features’ contribution, works in other types of histopathology slices. The classifier models, which can be used for the diagnosis of other cancer types based on immunochemical stained images and *in vivo* images (72, 73), remain to be explored and established.

**Figure 6:**
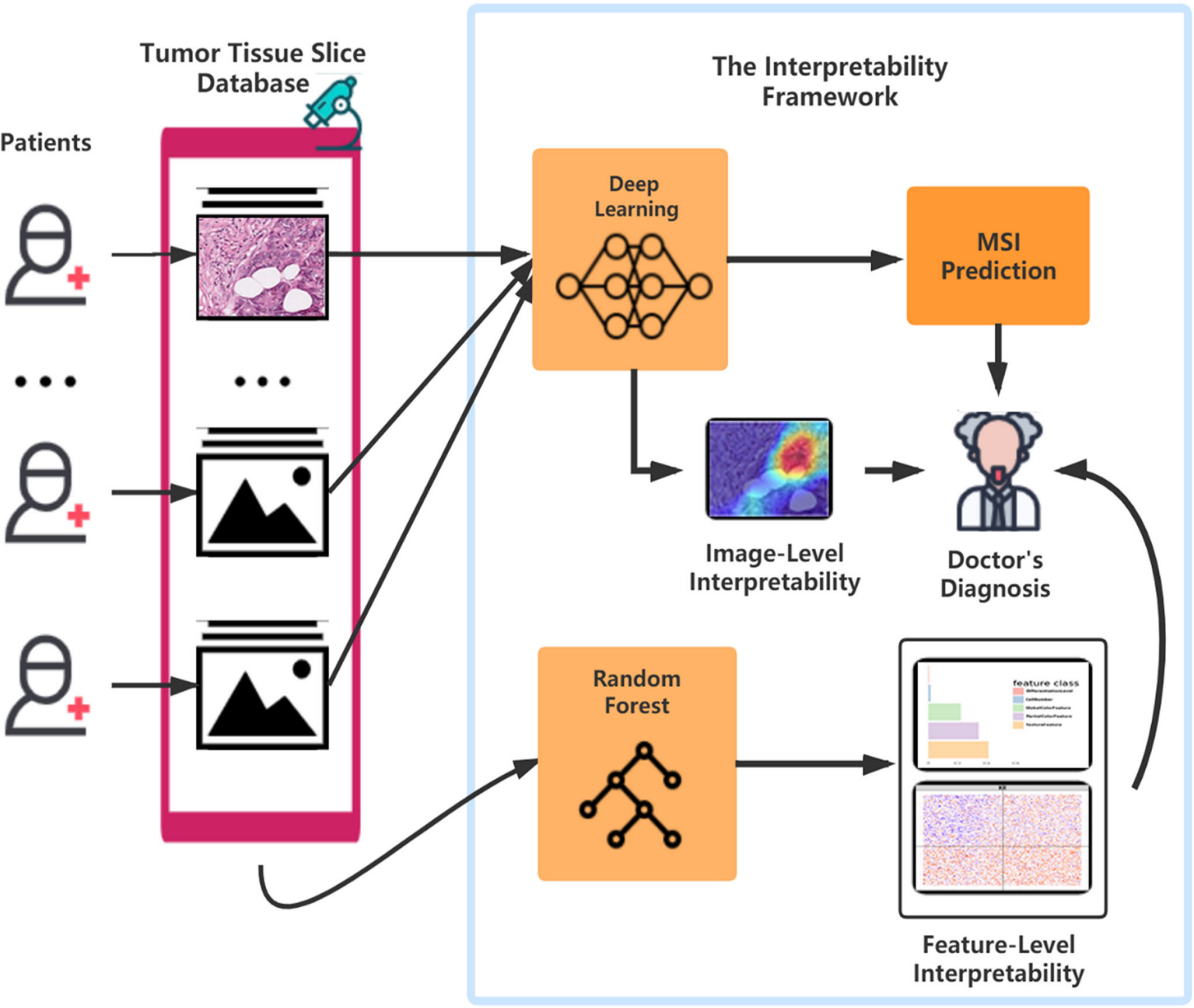
The flowchart of the pattern in which our framework can assist the doctor’s diagnosis. After surgery or biopsy, the embed cut H&E provided by each patient would go through MSI screening with deep learning. The doctor can make a critical diagnosis based on his insight combined with the prediction result and the deep learning model’s visualization. Meanwhile, with the amplifying of the H&E datasets, the random forest could develop a more precise and interpretable model, which helps the doctors detect MSI.

Further, our framework provides a positive feedback cycle in assisting pathologist’s diagnosis of MSI (**Figure 6**). Specifically, the localization map outputted by our DL models can help experts to narrow their focus on the specific region of the whole H&E slide, thereby contributing to a more accurate and apprehensible diagnosis with the prediction result of our model. The features’ distribution under our interpretable model can provide experts with more insight into analyzing the slices of MSI and MSS from clinical perspectives. Further, considering the similar feature distribution pattern in three datasets that we used, it is possible that, after running the same pipeline on MSI H&E slides under different cancer types, we can discover a generalization pattern behind them. After training on a larger dataset, the accuracy of the identification and the interpretability could improve, thereby contributing to accurate sample curation and treatment development of this aggressive cancer subtype.

## Data Availability Statement

Publicly available datasets were analyzed in this study. This data can be found here: https://zenodo.org/record/2530835.

## Author Contributions

JZ, WW, RL, and XW conceived and designed the study. JZ, WW, YZ, and YJ performed the statistical and computational analysis. Funding acquisition: RL, XW, and HZ, SL helped manuscript editing. All co-authors review and modify the manuscript and approving its final version.

## Funding

Wang's research is partially supported by NSFC(72171216, 71991474), the International Science & Technology cooperation program of Guangdong, China(2016B050502007), the Key Research and Development Program of Guangdong, China (2019B020228001), and Science and Technology Program of Guangzhou, China (202002030129). RL’s research is supported by the National Natural Science Foundation of China (81902381). HZ’s research is supported in part by U.S. National Institutes of Health (R01HG010171 and R01MH116527).

## Conflict of Interest

The authors declare that the research was conducted in the absence of any commercial or financial relationships that could be construed as a potential conflict of interest.

## Publisher’s Note

All claims expressed in this article are solely those of the authors and do not necessarily represent those of their affiliated organizations, or those of the publisher, the editors and the reviewers. Any product that may be evaluated in this article, or claim that may be made by its manufacturer, is not guaranteed or endorsed by the publisher.

## References

[1] Hause RJ Pritchard CC Shendure J Salipante SJ. Classification and Characterization of Microsatellite Instability Across 18 Cancer Types. Nat Med (2016) 22(11):1342. doi: 10.1038/nm.4191 27694933

[2] Popat S Hubner R Houlston RS. Systematic Review of Microsatellite Instability and Colorectal Cancer Prognosis. J Clin Oncol (2005) 23(3):609–18. doi: 10.1200/JCO.2005.01.086 15659508

[3] Cohen R Hain E Buhard O Guilloux A Bardier A Kaci R. Association of Primary Resistance to Immune Checkpoint Inhibitors in Metastatic Colorectal Cancer With Misdiagnosis of Microsatellite Instability or Mismatch Repair Deficiency Status. JAMA Oncol (2019) 5(4):551–5. doi: 10.1001/jamaoncol.2018.4942 PMC6459114 30452494

[4] Cheng DT Prasad M Chekaluk Y Benayed R Sadowska J Zehir A. Comprehensive detection of germline variants by MSK-IMPACT, A Clinical Diagnostic Platform for Solid Tumor Molecular Oncology and Concurrent Cancer Predisposition Testing. BMC Med Genomics (2017) 10(1):33. doi: 10.1186/s12920-017-0271-4 28526081 PMC5437632

[5] Suraweera N Duval A Reperant M Vaury C Furlan D Leroy K. Evaluation of Tumor Microsatellite Instability Using Five Quasimonomorphic Mononucleotide Repeats and Pentaplex PCR. Gastroenterology (2002) 123(6):1804–11. doi: 10.1053/gast.2002.37070 12454837

[6] Kautto EA Bonneville R Miya J Yu L Krook MA Reeser JW. Performance Evaluation for Rapid Detection of Pan-Cancer Microsatellite Instability With MANTIS. Oncotarget (2016) 8(5):7452–63. doi: 10.18632/oncotarget.13918 PMC5352334 27980218

[7] Li K Luo H Huang L Luo H Zhu X. Microsatellite Instability: A Review of What the Oncologist Should Know. Cancer Cell Int (2020) 20(1):16. doi: 10.1186/s12935-019-1091-8 31956294 PMC6958913

[8] Jenkins MA Hayashi S O’shea A-M Burgart LJ Smyrk TC Shimizu D. Pathology Features in Bethesda Guidelines Predict Colorectal Cancer Microsatellite Instability: A Population-Based Study. Gastroenterology (2007) 133(1):48–56. doi: 10.1053/j.gastro.2007.04.044 17631130 PMC2933045

[9] Greenson JK Huang S-C Herron C Moreno V Bonner JD Tomsho LP. Pathologic Predictors of Microsatellite Instability in Colorectal Cancer. Am J Surg Pathol (2009) 33(1):126. doi: 10.1097/PAS.0b013e31817ec2b1 18830122 PMC3500028

[10] Jass J. Classification of Colorectal Cancer Based on Correlation of Clinical, Morphological and Molecular Features. Histopathology (2007) 50(1):113–30. doi: 10.1111/j.1365-2559.2006.02549.x 17204026

[11] Alexander J Watanabe T Wu T-T Rashid A Li S Hamilton SR. Histopathological Identification of Colon Cancer With Microsatellite Instability. Am J Pathol (2001) 158(2):527–35. doi: 10.1016/S0002-9440(10)63994-6 PMC1850324 11159189

[12] Sagaert X Cutsem EV Tejpar S Prenen H Hertogh GD. MSI Versus MSS Sporadic Colorectal Cancers: Morphology, Inflammation, and Angiogenesis Revisited. J Clin Oncol (2014) 32(3):495–. doi: 10.1200/jco.2014.32.3_suppl.495

[13] Wong TY Bressler NM. Artificial Intelligence With Deep Learning Technology Looks Into Diabetic Retinopathy Screening. J Am Med Assoc (2016) 316(22):2366–7. doi: 10.1001/jama.2016.17563 27898977

[14] Serag A Ion-Margineanu A Qureshi H McMillan R Saint Martin M-J Diamond J. Translational AI and Deep Learning in Diagnostic Pathology. Front Med (2019) 6(185). doi: 10.3389/fmed.2019.00185 PMC6779702 31632973

[15] Iizuka O Kanavati F Kato K Rambeau M Arihiro K Tsuneki M. Deep Learning Models for Histopathological Classification of Gastric and Colonic Epithelial Tumours. Sci Rep (2020) 10(1):1–11. doi: 10.1038/s41598-020-58467-9 32001752 PMC6992793

[16] Bar Y Diamant I Wolf L Greenspan H. Deep Learning With non-Medical Training Used for Chest Pathology Identification. Med Imaging 2015: Computer-Aided Diagnosis; 2015: Int Soc Optics Photonics (2015) 9414. doi: 10.1117/12.2083124

[17] Kather JN Pearson AT Halama N Jäger D Krause J Loosen SH. Deep Learning can Predict Microsatellite Instability Directly From Histology in Gastrointestinal Cancer. Nat Med (2019) 25(7):1054–6. doi: 10.1038/s41591-019-0462-y PMC7423299 31160815

[18] Towards Trustable Machine Learning. Nat Biomed Eng (2018) 2(10):709–10. doi: 10.1038/s41551-018-0315-x 31015650

[19] Stiglic G Kocbek P Fijacko N Zitnik M Verbert K Cilar L. Interpretability of Machine Learning-Based Prediction Models in Healthcare. Wiley Interdiscip Reviews-Data Min Knowledge Discovery (2020) 10(5):e1379. doi: 10.1002/widm.1379

[20] Murdoch WJ Singh C Kumbier K Abbasi-Asl R Yu B. Definitions, Methods, and Applications in Interpretable Machine Learning. Proc Natl Acad Sci (2019) 116(44):22071–80. doi: 10.1073/pnas.1900654116 PMC6825274 31619572

[21] Jacobson NC Bentley KH Walton A Wang SB Fortgang RG Millner AJ. Ethical Dilemmas Posed by Mobile Health and Machine Learning in Psychiatry Research. Bull World Health Organ (2020) 98(4):270. doi: 10.2471/BLT.19.237107 32284651 PMC7133483

[22] Schaumberg AJ Juarez-Nicanor WC Choudhury SJ Pastrián LG Pritt BS Pozuelo MP. Interpretable Multimodal Deep Learning for Real-Time Pan-Tissue Pan-Disease Pathology Search on Social Media. Modern Pathol (2020) 1–17. doi: 10.1038/s41379-020-0540-1 PMC7581495 32467650

[23] Vellido A. Societal Issues Concerning the Application of Artificial Intelligence in Medicine. Kidney Dis (2019) 5(1):11–7. doi: 10.1159/000492428 PMC6388581 30815459

[24] Piano SL. Ethical Principles in Machine Learning and Artificial Intelligence: Cases From the Field and Possible Ways Forward. Humanities Soc Sci Commun (2020) 7(1):1–7. doi: 10.1057/s41599-020-0501-9

[25] Elshawi R Al-Mallah MH Sakr S. On the Interpretability of Machine Learning-Based Model for Predicting Hypertension. BMC Med Inf decision making (2019) 19(1):146. doi: 10.1186/s12911-019-0874-0 PMC6664803 31357998

[26] Lee E Choi J-S Kim M Suk H-I. Initiative AsDN. Toward an Interpretable Alzheimer’s Disease Diagnostic Model With Regional Abnormality Representation *via* Deep Learning. NeuroImage (2019) 202:116113. doi: 10.1016/j.neuroimage.2019.116113 31446125

[27] Network CGA. Comprehensive Molecular Characterization of Human Colon and Rectal Cancer. Nature (2012) 487(7407):330. doi: 10.1038/nature11252 22810696 PMC3401966

[28] Network CGAR. Comprehensive Molecular Characterization of Gastric Adenocarcinoma. Nature (2014) 513(7517):202–9. doi: 10.1038/nature13480 PMC4170219 25079317

[29] Liu Y Sethi NS Hinoue T Schneider BG Cherniack AD Sanchez-Vega F. Comparative Molecular Analysis of Gastrointestinal Adenocarcinomas. Cancer Cell (2018) 33(4):721–35.e8. doi: 10.1016/j.ccell.2018.03.010 29622466 PMC5966039

[30] Bailey MH Tokheim C Porta-Pardo E Sengupta S Bertrand D Weerasinghe A. Comprehensive Characterization of Cancer Driver Genes and Mutations. Cell (2018) 173(2):371–85.e18. doi: 10.1016/j.cell.2018.02.060 29625053 PMC6029450

[31] Macenko M Niethammer M Marron JS Borland D Woosley JT Guan X. A Method for Normalizing Histology Slides for Quantitative Analysis. In Boston: 2009 IEEE International Symposium on Biomedical Imaging: From Nano to Macro, vol. 2009. IEEE (2009). p. 1107–10.

[32] He K Zhang X Ren S Sun J. Deep Residual Learning for Image Recognition. In: Las Vegas Proceedings of the IEEE conference on computer vision and pattern recognition, vol. 2016. IEEE (2016). p. 770–8.

[33] Robin X Turck N Hainard A Tiberti N Lisacek F Sanchez J-C. pROC: An Open-Source Package for R and S+ to Analyze and Compare ROC Curves. BMC Bioinf (2011) 12(1):77. doi: 10.1186/1471-2105-12-77 PMC3068975 21414208

[34] Wickham H. Ggplot2: Elegant Graphics for Data Analysis. springer, New York (2016).

[35] Schneider CA Rasband WS Eliceiri KW. NIH Image to ImageJ: 25 Years of Image Analysis. Nat Methods (2012) 9(7):671–5. doi: 10.1038/nmeth.2089 PMC5554542 22930834

[36] Van der Walt S Schönberger JL Nunez-Iglesias J. Scikit-Image: Image Processing in Python. PeerJ (2014) 2:e453. doi: 10.7717/peerj.453 25024921 PMC4081273

[37] Linden MA Sedgewick GJ Ericson M. An Innovative Method for Obtaining Consistent Images and Quantification of Histochemically Stained Specimens. J Histochem Cytochem (2015) 63(4):233–43. doi: 10.1369/0022155415568996 PMC4374058 25575568

[38] Kuru K. Optimization and Enhancement of H&E Stained Microscopical Images by Applying Bilinear Interpolation Method on Lab Color Mode. Theor Biol Med Model (2014) 11(1):1–22. doi: 10.1186/1742-4682-11-9 24502223 PMC3923735

[39] Ruifrok AC Johnston DA. Quantification of Histochemical Staining by Color Deconvolution. Analytical quantitative cytol Histol (2001) 23(4):291–9. 11531144

[40] Yi F Huang J Yang L Xie Y Xiao G. Automatic Extraction of Cell Nuclei From H&E-Stained Histopathological Images. J Med Imaging (2017) 4(2):027502. doi: 10.1117/1.JMI.4.2.027502 PMC5478972 28653017

[41] Haralick RM Shanmugam K Dinstein I. Textural Features for Image Classification. IEEE Trans Systems Man Cybernetics (1973) SMC-3(6):610–21. doi: 10.1109/TSMC.1973.4309314

[42] Azevedo Tosta TA de Faria PR Neves LA do Nascimento MZ. Evaluation of Statistical and Haralick Texture Features for Lymphoma Histological Images Classification. Comput Methods Biomechanics Biomed Engineering: Imaging Visualization (2021) 1–12. doi: 10.1080/21681163.2021.1902401

[43] McLachlan GJ Peel D. Finite Mixture Models. John Wiley & Sons, New York (2004).

[44] Yuen H Princen J Illingworth J Kittler J. Comparative Study of Hough Transform Methods for Circle Finding. Image Vision computing (1990) 8(1):71–7. doi: 10.1016/0262-8856(90)90059-E

[45] Bankhead P Loughrey MB Fernández JA Dombrowski Y McArt DG Dunne PD. QuPath: Open Source Software for Digital Pathology Image Analysis. Sci Rep (2017) 7(1):1–7. doi: 10.1038/s41598-017-17204-5 29203879 PMC5715110

[46] Pedregosa F Varoquaux G Gramfort A Michel V Thirion B Grisel O. Scikit-Learn: Machine Learning in Python. J Mach Learn Res (2011) 12:2825–30.

[47] Bishop CM. Pattern Recognition and Machine Learning. (2006) 128(9):326–345.

[48] McCullagh P Nelder JA. In: London Generalized Linear Models. Routledge (2019).

[49] Wen Z Shi J Li Q He B Chen J. ThunderSVM: A Fast SVM Library on GPUs and CPUs. J Mach Learn Res (2018) 19(1):797–801.

[50] Breiman L. Random Forests. Mach Learn (2001) 45(1):5–32. doi: 10.1023/A:1010933404324

[51] Ishwaran H Kogalur UB Gorodeski EZ Minn AJ Lauer MS. High-Dimensional Variable Selection for Survival Data. J Am Stat Assoc (2010) 105(489):205–17. doi: 10.1198/jasa.2009.tm08622

[52] Paluszynska A Biecek P. Randomforestexplainer: Explaining and Visualizing Random Forests in Terms of Variable Importance. R Package Version 09. (2017).

[53] Wiener A. Classification and Regression by Randomforest. R News (2002) 2:18–22.

[54] Meyes R Lu M de Puiseau CW Meisen T. Ablation Studies in Artificial Neural Networks. arXiv preprint arXiv:190108644 (2019).

[55] Sheikholeslami S Meister M Wang T Payberah AH Vlassov V Dowling J. AutoAblation: Automated Parallel Ablation Studies for Deep Learning. In: New York Proceedings of the 1st Workshop on Machine Learning and Systems, vol. 2021. ACM (2021). p. 55–61.

[56] Du L. How Much Deep Learning Does Neural Style Transfer Really Need? An Ablation Study. In: New York Proceedings of the IEEE/CVF Winter Conference on Applications of Computer Vision, vol. 2020. AMC (2020). p. 3150–9.

[57] Selvaraju RR Cogswell M Das A Vedantam R Parikh D Batra D. Grad-Cam: Visual Explanations From Deep Networks via Gradient-Based Localization. In: Venice Proceedings of the IEEE International Conference on Computer Vision, vol. 2017. Kluwer Academic Publishers (2017). p. 618–26.

[58] Cheng J Han Z Mehra R Shao W Cheng M Feng Q. Computational Analysis of Pathological Images Enables a Better Diagnosis of TFE3 Xp11. 2 Translocation Renal Cell Carcinoma. Nat Commun (2020) 11(1):1–9. doi: 10.1038/s41467-020-15671-5 32286325 PMC7156652

[59] Echle A Laleh NG Schrammen PL West NP Trautwein C Brinker TJ. Deep Learning for the Detection of Microsatellite Instability From Histology Images in Colorectal Cancer: A Systematic Literature Review. ImmunoInformatics (2021) 100008. doi: 10.1016/j.immuno.2021.100008

[60] Biau G Scornet E. A Random Forest Guided Tour. Test (2016) 25(2):197–227. doi: 10.1007/s11749-016-0481-7

[61] Denisko D Hoffman MM. Classification and Interaction in Random Forests. Proc Natl Acad Sci (2018) 115(8):1690–2. doi: 10.1073/pnas.1800256115 PMC5828645 29440440

[62] Hao N Zhang HH. Interaction Screening for Ultrahigh-Dimensional Data. J Am Stat Assoc (2014) 109(507):1285–301. doi: 10.1080/01621459.2014.881741 PMC4224119 25386043

[63] Cordell HJ. Detecting Gene–Gene Interactions That Underlie Human Diseases. Nat Rev Genet (2009) 10(6):392–404. doi: 10.1038/nrg2579 19434077 PMC2872761

[64] Zhao Y Chung M Johnson BA Moreno CS Long Q. Hierarchical Feature Selection Incorporating Known and Novel Biological Information: Identifying Genomic Features Related to Prostate Cancer Recurrence. J Am Stat Assoc (2016) 111(516):1427–39. doi: 10.1080/01621459.2016.1164051 PMC5394568 28435175

[65] Schumacher TN Schreiber RD. Neoantigens in Cancer Immunotherapy. Science (2015) 348(6230):69–74. doi: 10.1126/science.aaa4971 25838375

[66] Le DT Durham JN Smith KN Wang H Bartlett BR Aulakh LK. Mismatch Repair Deficiency Predicts Response of Solid Tumors to PD-1 Blockade. Science (2017) 357(6349):409–13. doi: 10.1126/science.aan6733 PMC5576142 28596308

[67] Lee C-H Yelensky R Jooss K Chan TA. Update on Tumor Neoantigens and Their Utility: Why It Is Good to Be Different. Trends Immunol (2018) 39(7):536–48. doi: 10.1016/j.it.2018.04.005 PMC7954132 29751996

[68] McGrail DJ Garnett J Yin J Dai H Shih DJH Lam TNA. Proteome Instability Is a Therapeutic Vulnerability in Mismatch Repair-Deficient Cancer. Cancer Cell (2020) 37(3):371–86.e12. doi: 10.1016/j.ccell.2020.01.011 32109374 PMC7337255

[69] Braun M Menon R Nikolov P Kirsten R Petersen K Schilling D. The HOPE Fixation Technique - a Promising Alternative to Common Prostate Cancer Biobanking Approaches. BMC Cancer (2011) 11(1):511. doi: 10.1186/1471-2407-11-511 22151117 PMC3248383

[70] Chang S-C Lan Y-T Lin P-C Yang S-H Lin C-H Liang W-Y. Patterns of Germline and Somatic Mutations in 16 Genes Associated With Mismatch Repair Function or Containing Tandem Repeat Sequences. Cancer Medicine (2020) 9(2):476–86. doi: 10.1002/cam4.2702 PMC6970039 31769227

[71] Yamashita R Long J Longacre T Peng L Berry G Martin B. Deep Learning Model for the Prediction of Microsatellite Instability in Colorectal Cancer: A Diagnostic Study. Lancet Oncol (2021) 22(1):132–41. doi: 10.1016/S1470-2045(20)30535-0 33387492

[72] Xu Y Li C Lu S Wang Z Liu S Yu X. Design of a Metallacycle-Based Supramolecular Photosensitizer for *In Vivo* Image-Guided Photodynamic Inactivation of Bacteria. Angewandte Chemie (2022) 134(5):e202110048. doi: 10.1002/anie.202110048 34806264

[73] Xu Y Li C Lu S Wang Z Liu S Yu X. Construction of Emissive Ruthenium (II) Metallacycle Over 1000 Nm Wavelength for *In Vivo* Biomedical Applications. Nat Commun (2022) 13(1):1–13. doi: 10.1038/s41467-022-29572-2 35422104 PMC9010459

